# Intimate Partner Violence in the Golden Age: Systematic Review of Risk and Protective Factors

**DOI:** 10.3389/fpsyg.2018.01595

**Published:** 2018-09-04

**Authors:** Eva Gerino, Angela M. Caldarera, Lorenzo Curti, Piera Brustia, Luca Rollè

**Affiliations:** Department of Psychology, University of Torino, Torino, Italy

**Keywords:** golden age, IPV, risk factors, protective factors, aging

## Abstract

Intimate partner violence (IPV) is identifiable as a major public health concern worldwide. The international literature highlights how this phenomenon is complex and transversal to all age groups. While the global population is becoming older, the scientific research about risk and protective factors related to IPV in the golden age is diverse, and the different findings of the various studies have not been systematized so far. Thus, in this systematic review, we aim to analyze the scientific studies that investigate the risk and the protective factors of violent dynamics between elderly couples. From the perspective of the theoretical frameworks and the methodological approaches used, we present the main conceptual themes that emerge. Following the guidelines of the Preferred Reporting Items for Systematic Reviews and Meta-Analyses statement, we review the articles that report the analyses of protective and risk factors of IPV perpetration. Our results indicate social support, help-seeking behavior, and the availability of community-based services addressing the issues of abuse as the main protective factors. The risk factors are related to economic conditions, belonging to an ethnic minority, cognitive or physical impairment, other conditions associated with cultural background and relational dynamics, such as intrapartner dependence and intergenerational transmission of violence and trauma, and caregiving stress. We discuss possible future directions of research to improve the understanding of IPV in the elderly population and the implications for the development of intervention policies at preventive and supportive levels.

## Introduction

Intimate partner violence (IPV) refers to violence between couples. The World Health Organization (WHO, 2012) defined it as “any behavior within an intimate relationship that causes physical, psychological or sexual harm to those in the relationship” (p. 1), including acts of physical and sexual violence, emotional-psychological abuse, and controlling behaviors. It is important to distinguish IPV from domestic violence (DV), a comprehensive term that includes many types of domestic abuse, such as child and elderly abuse in a household. The term “intimate partner” indicates that violence can be perpetrated by both men and women, regardless of age, marital status, or sexual orientation (Archer, 2000; Capaldi et al., 2007; Ali et al., 2016).

In their recent review, Ali et al. (2016) find different classifications of IPV in the scientific literature. Their work outlines three main perspectives used to classify IPV, according to the types of (1) abuse, (2) violence, or (3) perpetrators. Regarding the types of abuse, WHO (2002) describes physical, sexual, and psychological categories.

As for the distinction according to the type of violence, Ali et al. (2016) report two classifications. The first is proposed by Johnson and Ferraro (2000), who classify five qualitatively different types of IPV: coercive controlling violence (CCV), violent resistance, situational couple violence (SCV), mutual violent control violence, and separation-instigated violence. CCV is described as “a pattern of control and manipulation by a partner against their intimate partner” (Ali et al., 2016, p. 18), where the coercive partner may use one or a combination of behaviors, such as intimidation, coercion, control, and physical violence, to keep the partner under control. A victim shows violent resistance to violence from a coercive controlling partner. SCV is “defined as the type of violence between partners when an individual can be violent and non-controlling in a relationship with a nonviolent partner or a violent but non-controlling partner” (Ali et al., 2016, p. 18). Mutual violent control violence occurs when both partners are violent and controlling toward each other (Ali et al., 2016, p. 19). Separation-instigated violence occurs between partners who are in the process of separation.

Ali et al. (2016) refer to the second classification as the “Johnston Typology” (Johnston and Campbell, 1993). Johnston and Campbell distinguish among IPV types based on the motivations for the use of violence and outline the categories of *episodic male battering, separation-engendered violence, male controlling interactive violence*, and *psychotic and paranoid reactions*.

Regarding the classification of IPV according to the types of perpetrators, the Authors find that it encompasses different approaches. These range from gender to the perpetrator's psychopathology (Holtzworth-Munroe and Meehan, 2004) or physiological activation and emotional arousal (Jacobson and Gottman, 1998) to the type of violence understood as a behavioral response (*generalized violent behavior, frustration response*, and *defensive behavior*; Miller and Meloy, 2006).

In addition to the complexity of the many ways of categorizing the construct, IPV presents significant variations across the life span from adolescence to young adulthood (Johnson et al., 2015) and to older age (Policastro and Finn, 2017). Specifically, senior years comprise a critical stage of life, where IPV has particular implications for intervention strategies (Roberto et al., 2014).

The United Nations (2017) reports that the number of people over 60 years old more than doubled (962 million worldwide in 2017 vs. 382 million in 1980), and it is expected to become twice larger again by 2050. In the light of such increase, the study on events strongly related to physical and mental health in older age becomes crucial (Gerino et al., 2017). However, to our best knowledge, a systematic review about studies on risk and protective factors is still missing.

Many studies indicate the difficulty of obtaining clear figures about the prevalence of IPV among the general population (Devries et al., 2013). The magnitude of IPV is also underestimated (Crockett et al., 2015). Such difficulty is more evident when examining IPV in old age. For example, Policastro and Finn (2017) note that it is possible to observe IPV occurring among the elderly in two ways—either as IPV on growing old or as a new experience of violence, initiated after the partners have reached their older years. Drawing data from the National Elder Mistreatment Study (a survey of a representative sample of older adults from the US), the two researchers find that 1.7% of the participants report experiencing physical violence after the age of 60, and 3.7% report experiencing emotional coercive controlling behavior by an intimate partner. However, Policastro and Finn (2017) acknowledge the heterogeneity of the prevalence data, mentioning, among many others, the study of Acierno et al. (2009), who find that around 10% of the participant elders have experienced a form of abuse and/or neglect, and for over half of the physical mistreatment cases, the partners are reported as the perpetrators. In the sample recruited for their study, Rosay and Mulford (2017) show that 22.2% of the elderly victims reporting psychological abuse have been assaulted by an intimate partner and likewise for 27.4% reporting physical abuse.

Being involved in physical and sexual IPV, as either a victim or a perpetrator, is negatively associated with physical and mental health across the life span (Costa et al., 2015). IPV has greater health consequences for older women (Crockett et al., 2015) and a strong impact on emotional wellbeing and mental health (McGarry et al., 2016), being related to feelings of greater “worthlessness” or a loss of a sense of identity over time.

Given the aging of the global population mentioned above (United Nations, 2017), the study on risk and protective factors related to IPV in old age is one of the most important strategies for planning prevention programs in communities. However, to date, the scientific literature is varied in scope and content, presenting interesting and heterogeneous data, which need to be systematized.

## Aims

Our study aims to present an up-to-date overview of risk and protective factors related to IPV in the golden age, focusing on the following:

1. analyzing the progress of studies across the years,
2. highlighting the presence of theoretical models about risk and protective factors, and
3. identifying future directions for research.

## Methods

### Data source and search strategy

We followed the guidelines of the Preferred Reporting Items for Systematic Reviews and Meta-Analyses (PRISMA) statement's rationale (Moher et al., 2009). PubMed and EBSCO databases (PsycArticles, PsycInfo, eBook Collection, CINAHL Complete, Education Source, Family Studies Abstracts, Gender Studies Database, Race Relation Abstracts, Social Sciences Abstracts, Sociology Source Ultimate, Urban Studies Abstracts, and Violence and Abuse Abstracts) were searched by browsing titles, abstracts, and full texts to find eligible studies published in English, from the beginning to March 2018, with the keywords (IPV OR intimate partner violence) AND (aging OR older OR elder OR seniors OR golden age). Considering the recent development of this research domain, we did not insert time limits. The two independent reviewers' search on EBSCO yielded 986 results; 85 met the criteria and were selected. A second search on PubMed was performed to identify other papers; from the 597 results found (with a significant overlap with the previous search), only four papers met the criteria and were selected, totaling 89 papers screened by title and abstract. Subsequently, all 89 papers were screened by text, and from these, 58 papers provided specific information about IPV and aging. Eventually, the last phase entailed the selection of the papers specifically concerning risk and protective factors. Among those, 30 papers included the analysis of risk factors, while only eight included the analysis of protective factors. Considering that six papers included both risk and protective factors (Gil et al., 2015; Guedes et al., 2015; Yan, 2015; Roh et al., 2016; Souto et al., 2016; Teresi et al., 2016), the papers dealing with risk and protective factors that were included in this systematic review totaled 32.

Since we used databases containing peer-reviewed international journals, most of the studies included in the research were written in English. This implies that the research could miss hypothetical studies in other languages or those studies not published in peer-reviewed international journals. This issue is addressed in the Limitations section.

## Inclusion and exclusion criteria

The first three inclusion criteria for the papers were (a) the presence of the IPV construct, (b) an older population (average ≥ 55 years), and (c) the English language. We took the age of 55 as the cutoff because it is the lowest cutoff used in the literature (Zink et al., 2006; Poole and Rietschlin, 2012; Sood et al., 2016) to separate adulthood from the golden age; therefore, it is the most inclusive, except the study of Paranjape et al. (2009), using the age of 50. The cutoff age seems related to countries' specific demographic characteristics; for example, Adjukovic et al. (2009) use 65 as the cutoff because it is the age of retirement in their Croatian sample. We also included papers focusing on constructs connected to IPV, such as domestic violence, family violence, and elder abuse, when related to violence between partners and spouses, excluding those unrelated to intimate partner situations. Both qualitative and quantitative articles were selected in our attempt to show different approaches and methodologies regarding the subject matter. We included quantitative papers with different and cross-cultural kinds of populations. All the papers that emerged from the search with no direct link to IPV among older populations were excluded. Subsequently, we selected the papers that aimed to investigate the risk and the protective factors in this specific sample in order to systematize them in a table.

**Figure 1:**
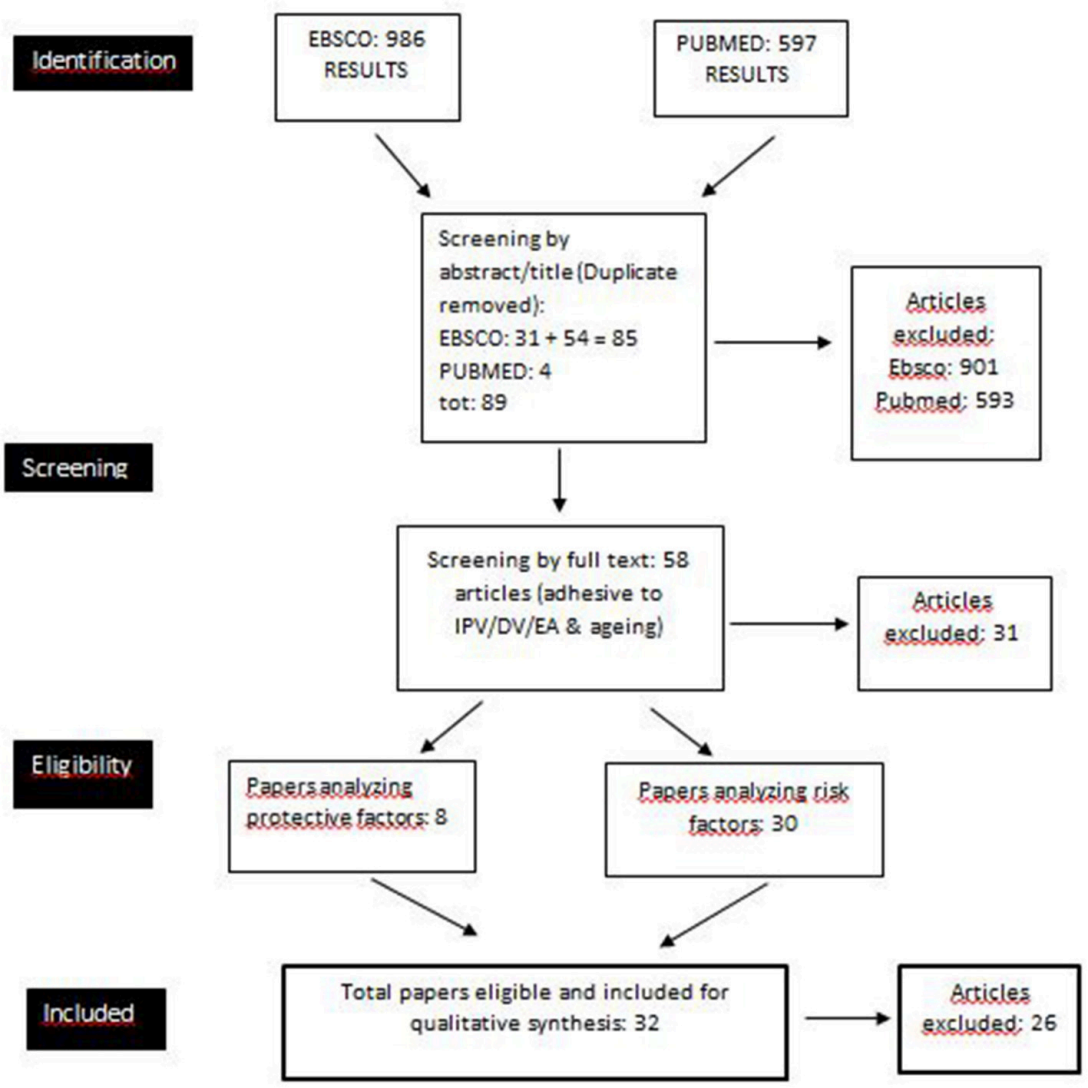
Flow diagram of the selection procedure.

**Figure 2:**
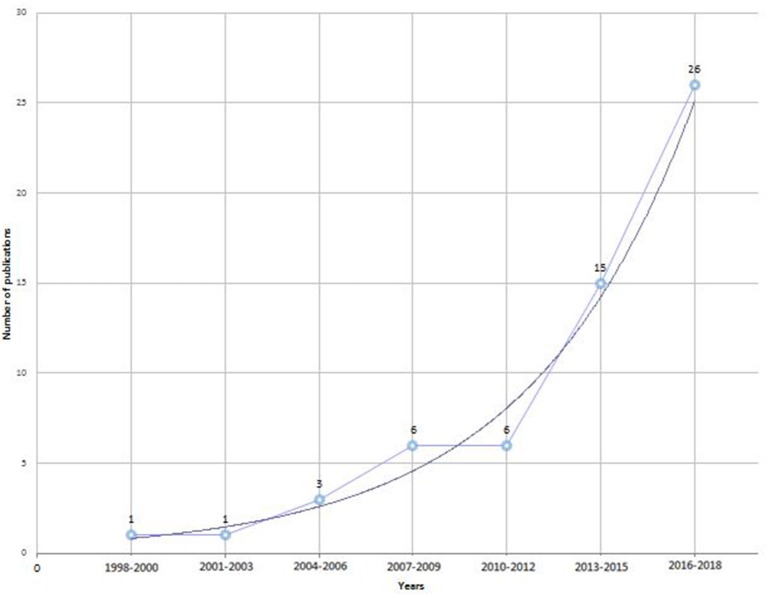
Diagram of the studies retrived for the review: number of publications across time.

The review process is summarized in Figure 1, while Figure 2 shows the growing number of published studies on the issue over time.

## Results

The papers about risk and protective factors of IPV in the senior years are derived mainly from North America (9 from the US, including those dealing with ethnic minorities; 3 from Canada, and 1 from a sample of the North American indigenous population). It is remarkable that an article involves study participants of Korean descent in California and another is about Portuguese immigrants in Canada. Several papers come from Asia (1 systematic review about Asia, 4 from China, and 2 from Korea), while Europe seems less represented (1 article each from Croatia, Germany, and Albania and 2 from Portugal). IPV and aging in South America have also been studied in two samples (Colombia and Brazil). It should be noted that the Albanian, Colombian, Brazilian, and Canadian) samples have been studied and reported in the same paper (Guedes et al., 2015).

### Methodological issues: research methods and assessment measures used in the retrieved papers

The papers included in this systematic review present several methodological and theoretical differences, partially due to the coexistence of many branches and disciplines involved in these issues (e.g., nursery, criminology, psychology, social services). Furthermore, the research conducted in many areas of the world (e.g., Southeast Asia, North America, Europe, Africa) and in different social contexts (e.g., rural areas or immigrants and ethnic minorities) shows cultural differences although the selected construct (IPV) has been defined in the same way in cross-cultural papers. Among these, the most studied contexts and ethnic groups in the literature about IPV and aging are the Western (US) rural areas (Teaster et al., 2006; Brossoie and Roberto, 2015; Weeks et al., 2016; Roberto and McCann, 2018) and Asian elders (both residents in Southeast Asia and immigrants in Western countries) (Yan and Chan, 2012; Yan, 2015; Yan et al., 2015; Cheung et al., 2016; Han et al., 2017; Nam and Lincoln, 2017; Qin and Yan, 2018). The reason why so much literature has been produced about these cultures is made clearer later in this article. Considering all the papers (58 selected from the databases and noted in Figure 1), the most used assessment measurement adopted in quantitative research to investigate IPV among older people is the Conflict Tactics Scale (Straus, 1979), sometimes in a revised form (Sormanti et al., 2004; Sormanti and Shibusawa, 2008; Liles et al., 2012; Yan and Chan, 2012; Stöckl and Penhale, 2015; Roh et al., 2016; Nam and Lincoln, 2017). Other measurement tools, mainly used to investigate constructs similar to IPV, such as domestic violence and elder abuse, include the Multidimensional Measure of Emotional Abuse Questionnaire (MMEAQ, Hazrati et al., 2017), the Hurt, Insulted, Threatened with harm, and Screamed scale (Guedes et al., 2015; HITS, Miszkurka et al., 2016), or the Family Violence Against Older Women scale (FVOW, Paranjape et al., 2009). Researchers also utilize national and clinical services' databases to investigate the prevalence and correlates of IPV/domestic violence among elders (Salari and Maxwell, 2016; Sood et al., 2016; Policastro and Finn, 2017; Rosay and Mulford, 2017). The quantitative research papers are mainly cross-sectional; the difficulties in conducting longitudinal studies are probably due to the novelty of the issues, also caused by the victims' historical tendency to conceal their situation (McGarry et al., 2016). In qualitative research, the most used tools are in-depth face-to-face interviews (Zink et al., 2006; Tetterton and Famsworth, 2011; Band-Winterstein, 2013, 2015; Eisikovits and Band-Winterstein, 2015; Yan, 2015; Weeks et al., 2016) and semi-structured interviews (Roberto and McCann, 2018). Band-Winterstein's papers are characterized by a phenomenological approach and discourse analysis. In qualitative studies, focus groups (Cianelli et al., 2013; Gil et al., 2015) have been used both to analyze and describe the phenomenon and to improve the participants' mental health, from a research-action and a community-ecological perspective (Brossoie and Roberto, 2015). The usefulness of including both qualitative and quantitative research is that the latter provides the prevalence and the risk/protective factors of the IPV phenomenon among the elderly, whereas the former can be helpful in explaining, somewhat clinically, the associations among the factors analyzed in quantitative research. A meta-ethnographic synthesis of qualitative evidence (McGarry et al., 2016, p. 2187) analyzes the following three fundamental themes of IPV in late life, aiming to show the variability of the phenomenon: (a) “unspoken and hidden” (b) “changing nature of IPV over time,” and (c) “longevity of abuse.” The first dimension refers to the hiddenness of the violence and the victims' inability to disclose and talk about IPV. The third dimension highlights the importance of the difference in the longevity of abuse; “older women may either have experienced IPV over the course of a long-term partnership or as a result of entering into new relationships later in life” (McGarry et al., 2016, p. 2188). The “changing nature of IPV over time” is also emphasized because many older women experience changes in the violence, for example, the transition from physical to psychological abuse in the relationship.

Beyond the methodological issues, it is important to mention the different theoretical and conceptual frameworks emerging from the research about IPV and aging. The first theoretical model to be produced is Sev'er's (2009) trilevel conceptual model of elder abuse (which includes IPV as a subtheme). This model aims to show the complexity of the issues involved in this phenomenon, including both personal and social characteristics and structural inhibitors and accelerators, highlighting the potentially different effects (inhibiting-accelerating) of specific circumstances. As shown by Roberto et al. (2014), two conceptual frameworks also emerge from the literature about IPV in the golden age—the feminist and the ecological models. The first model analyzes power dynamics in late-life relationships with a feminist lens. It aims to show the gender-biased structure of late-life families, where older women are victimized and repressed by structural elements temporally prior to feminist instances and fights (e.g., Roberto and McCann, 2018 [“feminist life course perspective”]; Weeks et al., 2016). The second model is guided by the idea that it is impossible to conceive of a phenomenon such as IPV in late life without considering multiple layers, including context, cultural and societal values, family, community, and formal and informal social support. This conceptual framework is mainly oriented to intervention and research-action; in this context, it is possible to find a phenomenological approach as well (e.g., Teaster et al., 2006; Bonomi et al., 2007; Band-Winterstein, 2012, 2013, 2015; Poole and Rietschlin, 2012; Eisikovits and Band-Winterstein, 2015). More recently, Teresi et al. (2016) provide a conceptual framework to analyze elder abuse and IPV in late life. This complex ecological-cybernetical model includes stressful events (that produce symptoms); social structure and environment; presence or absence of primary and secondary prevention; psychological, social, and financial resources; and presence or absence of precipitating conditions (e.g., dementing illness and psychiatric or neurological diagnosis).

Despite the presence of these conceptual frameworks, many quantitative studies move away from a non-theoretical perspective, preferring to show empirical data without inserting them in theory-oriented research (e.g., Sood et al., 2016; Rosay and Mulford, 2017). To sum up, it is possible to identify two methodological–conceptual axes (dimensions) in the research about IPV and aging, as follows:

- *Qualitative*–*quantitative dimension*. Quantitative studies are mainly cross-sectional and descriptive. Qualitative studies are mostly characterized by research-action and phenomenological approaches.
- *Conceptual*–*non-conceptual dimension*. The prevalence and correlation studies are mainly non-theoretical (or measurement based), while other kinds of papers adopt and claim a conceptual framework (generally feminist or ecological).

Although qualitative papers tend to be characterized by a theoretical framework, we prefer not to overlap the two dimensions. In fact, some quantitative articles are also theory oriented (e.g., Poole and Rietschlin, 2012; Miszkurka et al., 2016).

### Protective factors

The protective factors detected in IPV (see Table 1) during the senior years are reappraisal (1 paper), community (2), having friends (3), generally speaking, social support and networks (2), help-seeking behavior (1), protective interventions from childhood (1), self-esteem (1), coping strategies (life skills) (1), and eventually becoming an immigrant in Canada (1). Significantly, in all the literature about IPV in late life, only eight articles specifically deal with and analyze protective factors. Rather, it seems that research on this theme has proceeded in the direction of either identifying risk factors or extrapolating protective ones from the absence of the risk factors.

**Table 1:** Published studies on protective factors.

**References**	**Title**	**Type of paper**	**Sample or participants**	**Identified protective factors**
Zink et al., 2006	A lifetime of intimate partner violence	Research	38 women ≥55 years (US)	Reappraisal, community, friends
Liles et al., 2012	Prevalence and correlates of intimate partner violence among young, middle, and older women of Korean descentin California	Research (qualitative)	*N* = 592 Korean women	Social support downsized as a protective factor
Gil et al., 2015	Development of a culture sensitive prevalence study on older adults violence: qualitative methods contribution	Research (qualitative)	13 interviews with older adults victimized by spouse (*n* = 7), sons or daughters (*n* = 6). 4 focus groups (32 subjects). Portugal	Informal and formal social networks
Yan, 2015	Elder abuse and help-seeking behavior in elderly Chinese	Research (qualitative)	40 women (Hong Kong)	Help-seeking behavior
Guedes et al., 2015	Socioeconomic status, social relations and domestic violence (DV) against elderly people in Canada, Albania, Colombia and Brazil	Research	Data on socioeconomic status and social relations collected in 2012 from1,995 community-dwelling older adults in Canada, Colombia, Brazil, and Albania	Having friends: detected in developed countries, not observed in Latin American and Eastern European participants
Roh et al., 2016	Risk and protective factors for depressive symptoms among indigenous older adults: intimate partner violence (IPV) and social support	Research	*N* = 233 indigenous older adults (North America)	Social support protective of both IPV and depressive symptoms
Teresi et al., 2016	State of the science on prevention of elder abuse and lessons learned from child abuse and domestic violence prevention: toward a conceptual framework for research	Review	21 intervention programs on prevention of elder abuse	Interventions in the protection from violence since childhood can be interpreted as protective factors of IPV in late life. Generally speaking, self-esteem and coping strategies, supported by knowledge and life skills, can be targeted to develop interventions and change models. Resources include social determinants and sociodemographic variables, for example, financial resources; cultural factors, such as race/ethnicity and acculturation; knowledge and skills; and psychological resources, such as self-esteem and coping.
Souto et al., 2016	Intimate partner violence among older Portuguese immigrant women in Canada	Qualitative research (socio-phenomenological approach)	10 women ≥60 years	Becoming an immigrant in Canada

The protective factor, *par excellence*, emerging from the literature is social support (Zink et al., 2006; Gil et al., 2015; Roh et al., 2016), defined as comprising formal or informal social networks (community, friends, and social/protective services). Zink et al. (2006) also attach importance to the reappraisal of a victim's situation. The contribution of Zink et al. shows that all these factors can influence the effectiveness of actual coping strategies of IPV victims in late life. Social support seems to protect IPV victims from the pejorative loop. However, in another research on a particular sample (youngsters to older Korean immigrants and descendants in California) (Liles et al., 2012), social support does not emerge as a protective factor among women under the age of 40 but as a paradoxical risk factor (and it does not emerge as a protective factor for women over 55). This phenomenon is due to specific cultural values. For the authors, the traditional Korean values expressed in the social networks (mainly, patriarchal Confucianism) are not coherent with the protection of women's health from the adverse effects IPV. The relevance of the variability of cultural values to the effectiveness of the social network as a protective factor is also highlighted by Guedes et al. (2015), who find a protective effect of having friends in developed countries (where friends substitute for family ties) but not among Latin American and Eastern European participants. These findings are consistent with those of Souto et al. (2016) who identify “becoming an immigrant in Canada” (p. 12) as a protective factor. Although this could seem to be a powerful stressor that would trigger psychological and physical violence, the change of status (and state) actually allowed the battered women to be more protected by a different culture and system of formal and informal support.

Another protective factor emerging from the literature (Yan, 2015) is the help-seeking behavior of elderly victims of abuse and IPV. A recent paper (Teresi et al., 2016) analyzes elder abuse and IPV in late life in association with research on child maltreatment and abuse. Based on their collected data from studies about IPV across the life span, the authors claim that some intervention strategies could also be protective for elder people, such as legal programs; medical interventions; social services; training in violence prevention, assertiveness, and resistance; and skill enhancement and practice (even if these interventions are provided for children). This way, interventions in protection from violence since childhood can be interpreted as a protective factor for IPV in late life. Generally speaking, self-esteem and coping strategies, supported by knowledge and life skills, can be targeted to develop interventions and change models (Teresi et al., 2016).

### Risk factors

The risk factors emerging from the literature (see Table 2) are gender (7 papers), age (5), parental violence and intergenerational transmission of violence (7), low social support and isolation from the community (6), cognitive impairment, such as dementia and Alzheimer's disease (9), physical impairment (6), cultural values and factors (4), depressive symptoms (4), ethnic differences (3), immigration stress (1), unemployment and low income (3), personal factors, such as life stress (3), relational factors, such as living with an abusive partner (4), environmental factors, such as little privacy (3), verbal abuse (2), substance abuse by both perpetrator and victim (2), and caregiver stress (1). The research on IPV in the golden age provides much more literature about risk factors than protective ones. Despite this difference, most of the associations between predisposing factors and IPV in late life are still not completely explained, partly because of the inextricability of the relations between variables. For this reason, it seems useful to include papers where IPV is interpreted as a risk factor of severe symptoms, such as depression (e.g., Nam and Lincoln, 2017), to evaluate the circularity of the associations between other variables. In fact, a systematic review (Yan et al., 2015) reports depression as a risk factor of IPV in the elderly population, thus reversing the direction of the relation between the variables.

**Table 2:** Published studies on risk factors.

**References**	**Title**	**Type of paper**	**Sample or participants**	**Identified risk factor**
Sormanti et al., 2004	Considering HIV risk and intimate partner violence among older women of color: a descriptive analysis	Descriptive analysis	139 African American and Latin American women aged 50 and older receiving care in outpatient clinics of an urban medical center	HIV (risk and consequence)
Adjukovic et al., 2009	Family violence and health among elderly in Croatia	Research (cross-sectional retrospective study)	303 elder Croatian men and women	Female gender, although also men are victims of family violence, according to Croatian official criminal data.
Paranjape et al., 2009	Lifetime exposure to family violence: implications for the health status of older African American women	Quantitative research	158 African American women, aged > 50	Unemployment
Sev'er, 2009	More than wife abuse that has gone old: a conceptual model for violence against the aged in Canada and the US	Review	//	Female genderTri-conceptual model of IPV among elderly
Poole and Rietschlin, 2012	Intimate partner victimization among adults aged 60 and older: an analysis of the 1999 and 2004 general social survey	Descriptive Research	Canadian sample A weighted cross-sectional sample pooled from cycles 13 (1999) and 18 (2004) of Statistics Canada's General Social Survey	Personal, relational, and environmental factors
Liles et al., 2012	Prevalence and correlates of intimate partner violence among young, middle, and older women of Korean descentin California	Quantitative research	592 Korean women residents of California	Immigration stress strongly predictive of abuse in the oldest age group
Yan and Chan, 2012	Prevalence and correlates of intimate partner violence amongolder Chinese couples in Hong Kong	Quantitative research	Only participants aged 60 or above and married or cohabiting at the time of the interview. 937 cases (397 women and 540 men) extracted and included in analysis	Younger people among the “older” group Unemployment Substance abuse problem Traumatisation during childhood Past criminal history Low level of assertiveness Anger Management problem Low social support
Cianelli et al., 2013	Unique factors that place older Hispanic women at risk for HIV: intimate partner violence, machismo, and marianismo	Qualitative/quantitative research	5 focus groups (50 participants)	IPV involved in HIV (as risk and consequence)
Roberto et al., 2014	Intimate partner violence in late life: a review of the empirical literature	Empirical literature review	57 empirical sources	Fear, social isolation, cognitive and physical impairment
Yan et al., 2015	A systematic review of prevalence and risk factors for elder abuse in Asia	Systematic review	Articles included Chinese (PRC: 8, Taiwan: 3, Hong Kong: 4 articles and 1 report, US Chinese: 1); Indian (5 articles and 2 reports); Singaporean (2); Japanese (9); and Korean (Korea/South Korea: 7, US Korean: 5).	Low income, poor physical health, low cognitive functioning, absence of social support, depressive symptoms
Policastro et al., 2015	Conceptualizing crimes against older persons: elder abuse, domestic violence, white-collar offending, or just regular “old” crime	Descriptive analysis	Information collected from 750 protective services cases (the 250 most recent cases from each social services agency). FTotal: 294 cases	Gender, ethnic differences, Alzheimer's disease, psychiatric problems
Gil et al., 2015	Development of a culture sensitive prevalence study on older adults violence: qualitative methods contribution	Qualitative research	13 interviews with older adults victimized by spouse (*n* = 7), sons, or daughters (*n* = 6). 4 focus groups (totaling 32 participants) (Portugal)	Neglect, Caregiver stress and burden
Yan, 2015	Elder abuse and help-seeking behavior in elderly Chinese	Qualitative research	40 women (Hong Kong)	Intergenerational transmission of violence
Stöckl and Penhale, 2015	Intimate partner violence and its association with physical and mental health symptoms among older women in Germany	Quantitative research (cross-sectional design)	Data from a national representative survey of 10,264 German women aged 16 to 86	High levels of education (although the victims could use them to ask for protective services), little privacy
Guedes et al., 2015	Socioeconomic status, social relations and domestic violence (DV) against elderly people in Canada, Albania, Colombia and Brazil	Research	Data on socioeconomic status and social relations collected in 2012 from1,995 community-dwelling older adults in Canada, Colombia, Brazil, and Albania	Intergenerational conflicts and/or strains arising from caregiver roles may partially explain the negative impact of multigenerational living arrangements. No associations for low income and education (if adjusted for social support and living arrangements). The convoy framework asserts that the effect of social support varies by gender
Crockett et al., 2015	Survivors in the margins: the invisibility of violence against older women	Commentary	//	Negative associations between age and violence. Patriarchal valuesCulturesocial hierarchies (based on race, socioeconomic statuses, gender identity, sexual orientation)
Sood et al., 2016	Self-reported verbal abuse in 1300+ older women within a private, tertiary women's health clinic	Database research (Mayo Clinic, Minnesota)	1,389 women with a median age of 55 (range: 50–90)	Verbal abuse
Cheung et al., 2016	Intimate partner violence in late life: a case study of older Chinese women	Case study	2 Chinese women (aged over 60)	Cultural values
Roh et al., 2016	Risk and protective factors for depressive symptoms among indigenous older adults: intimate partner violence (IPV) and social support	Quantitative research	233 older indigenous people (North America)	Depressive symptomatology as risk and consequence
Beach et al., 2016	Screening and detection of elder abuse: research opportunities and lessons learned from emergency geriatric care, intimate partner violence, and child abuse	Review	Different sources: health care screenings, direct victim surveys, caregiver surveys, forensic analysis,	Disability, especially cognitive impairment, and sexual changes related to the aging process or cognitive impairment. Although IPV victimization rates for women decrease with age, the adverse physical and mental health outcomes associated with IPV are similar for younger and older women
Teresi et al., 2016	State of the science on prevention of elder abuse and lessons learned from child abuse and domestic violence prevention: toward a conceptual framework for research	Review	21 intervention programs on prevention of elder abuse	Social structure and the environment, including social support and living arrangements
Salari and Maxwell, 2016	Lethal intimate partner violence in later life; understanding measurements, strengths, and limitations of research	Descriptive quantitative research on databases:	U.S. Databases (as Bureau of Justice Statistics National Crime Victimization Survey)	Depressive symptomsAccess to firearmsPrevious attempted suicideMajor life stresses such as poor healthCoercive perpetrator with patriarchal attitude, misogyny, lack of empathyVictim isolationPrevious IPV incidents
Souto et al., 2016	Intimate partner violence among older Portuguese immigrant women in Canada	Qualitative study (socio-phenomenological approach)	10 women ≥60	Cultural beliefs about marriage
Miszkurka et al., 2016	Correlates of partner and family violence among older Canadians: a life-course approach	Quantitative research	Baseline data (2012) from two Canadian sites of the International Mobility in Aging Study (IMIAS) involving community-dwelling individuals aged 65 to 74. Participants in Kingston, Ontario (*N* = 398 total, *n* = 186 men, *n* = 12 women) and Saint-Hyacinthe, Quebec (*N* 401 total, *n* = 191 men, *n* = 210 women)	Gender, social isolation, substance abuse of perpetrator, mental and physical impairment, verbal abuse, poor quality of relations, childhood victimization
Policastro and Finn, 2017	Coercive control and physical violence in older adults	Data analysis	5,103 subjects (US)	Experiencing trauma, poor health, low levels of social support, and living alone are signs. Associated with increased risk of physical abuse
Han et al., 2017	Factors influencing beliefs about intimate partner violence among adults in South Korea	Cross-sectional descriptive study	466 older Koreans	Low education, assisting parental violence
Altman, 2017	A crime at any age: intimate partner abuse in later life	Review	//	Cognitive bias, dementia, being with an abusive partner, substance abuse, isolation from the community
Nam and Lincoln, 2017	Lifetime family violence and depression: the case of older women in South Korea	Quantitative research	525 older Korean women	IPV risk and factor for depressive symptoms
Rosay and Mulford, 2017	Prevalence estimates and correlates of elder abuse in the United States: The National Intimate Partner and Sexual Violence Survey	Quantitative research	2,185 subjects, aged ≥70 (National Intimate Partner and Sexual Violence Survey)	Functional impairment, difficulties with activities of daily living, low social support and income, prior trauma, poor health, race, gender
Santos et al., 2017	Psychological elder abuse: measuring severity levels or potential family conflicts?	Research (cross-sectional study)	1,123 subjects	Gender, age (group more at risk: women aged between 60 and 69). Cohabitation is a variable relevant only to abuse as assessed by the stricter measure (>10 times)
Qin and Yan, 2018	Common crime and domestic violence victimization of older Chinese in urban China: the prevalence and its impact on mental health and constrained behavior	Quantitative research	Representative sample of 453 older adults aged 60 or above recruited from Kunming, People's Republic of China, using multistage sampling method	Over half of the participants had a mental impairment. Experiences of common crime victimization and fear of domestic violence are linked to risk factors for impaired mental health

### Social-demographic characteristics: gender, age, and socioeconomic status

The most studied risk factor for IPV in late life is gender; the majority of the studies take for granted the association between elder abuse and IPV and their relevance to older women. We can state that for researchers, the involved population is primarily that of women. Some studies (Sev'er, 2009; Guedes et al., 2015; Policastro et al., 2015; Miszkurka et al., 2016; Santos et al., 2017) directly examine and show the prevalence of women as IPV victims although a study in the Croatian context indicates that older men can be victims of family violence as well (Adjukovic et al., 2009). The literature generally claims the necessity to adopt a gender-oriented approach (aligned with the feminist model) (e.g., Guedes et al., 2015), yet a recent review of the empirical literature about this topic (Roberto et al., 2014) highlights the absence of research about female-on-male violence.

Other risk factors associated with IPV in late life are related to demographic and non-demographic characteristics of victims. Age seems to be a relevant variable because it is negatively associated with IPV in the older population. The younger segment (aged 55–69) of the elder population is at higher risk of being involved in an IPV situation (both psychological and physical abuse). In contrast, older women (> 69) seem less at risk (Yan and Chan, 2012; Crockett et al., 2015; Beach et al., 2016; Santos et al., 2017), probably due to facts related to aging (e.g., the death of the abuser or separation/divorce) (Miszkurka et al., 2016). Race seems to be a risk factor of IPV in late life; in fact, older people (women) belonging to ethnic minorities seem more predisposed to IPV (Sormanti et al., 2004; Paranjape et al., 2009; Liles et al., 2012; Cianelli et al., 2013; Souto et al., 2016). The victims' unemployment (Paranjape et al., 2009; Yan and Chan, 2012) and low income (Guedes et al., 2015; Yan et al., 2015) are also risk factors for IPV. It is remarkable that low income and unemployment are generally more associated with females than with males. A low level of education seems to be a risk factor of IPV in late life (Han et al., 2017) although a high level of education is not necessarily a protective factor even if correlated with more victims' awareness about protective services (Stöckl and Penhale, 2015). In another research, no associations between low income and education and IPV are observed if adjusted for social support and living arrangements (Guedes et al., 2015). For these authors, the more relevant risk factor is the low level of support received from family members, as well as the isolation from the community that derives from it (Altman, 2017; Policastro and Finn, 2017).

### Mental and physical health

Another issue emerging from the literature is the high correlation between cognitive (e.g., Alzheimer's, neurological, and psychiatric diseases related to aging) and physical impairment and IPV in late life (Roberto et al., 2014; Yan et al., 2015; Beach et al., 2016; Miszkurka et al., 2016; Altman, 2017). This association could be interpreted both as IPV influencing mental and physical health (Qin and Yan, 2018) and as mental and physical health influencing IPV. Verbal abuse seems to be a risk factor and a predictor of physical abuse (Sood et al., 2016). Substance (particularly alcohol) abuse emerges as a risk factor of IPV among older couples (Liles et al., 2012; Miszkurka et al., 2016; Altman, 2017).

### Cultural factors

Cultural beliefs and, generally speaking, cultural and societal values, emerge as relevant risk factors in late-life IPV (Souto et al., 2016). In fact, IPV seems to be more prevalent among ethnic minorities (yet at risk of other factors), often marked by powerful stressors (e.g., immigration) and machistic-patriarchal values (Sormanti et al., 2004; Paranjape et al., 2009; Liles et al., 2012; Cianelli et al., 2013). Late-life IPV also occurs more frequently in contexts where feminist trends have not arrived, such as in rural areas (Brossoie and Roberto, 2015; Weeks et al., 2016; Roberto and McCann, 2018) and in Confucian Asia (Yan and Chan, 2012; Yan, 2015; Yan et al., 2015; Cheung et al., 2016; Han et al., 2017; Nam and Lincoln, 2017; Qin and Yan, 2018). If aging itself brings more vulnerabilities for victims, it is probable that women remain subject to IPV and abuse due to the same social norms that impose the gender hierarchy (Crockett et al., 2015). Generally, racism (even when introjected) and sexism are ideological risk factors for IPV in late life (Poole and Rietschlin, 2012). Another important risk factor analyzed in the literature is the intergenerational transmission of violence and trauma. In fact, experiencing trauma in early life seems to be a predictor of acting with (or receiving) violence in late life (Guedes et al., 2015; Yan, 2015; Miszkurka et al., 2016; Policastro and Finn, 2017; Rosay and Mulford, 2017). The literature shows the importance of paying attention to every variable at stake; personal, relational (e.g., dependence on the partner by a victim with an impairment), and environmental factors can play a determining role in the phenomenon (Poole and Rietschlin, 2012). In fact, the research claims that caregiver stress and burden (in this case, the caregiver is the partner or the spouse) can also be risk factors for IPV (Gil et al., 2015). To our best knowledge, there is no published research yet about the differences (and different risk factors) between “IPV grown old” and new experiences of IPV in later life (Cheung et al., 2016).

## Discussion

### Summary of main findings

#### Protective factors

This systematic review involving older romantic couples has made it possible to highlight how research has not yet clearly identified the protective factors for victims or couples in situations where violent dynamics are or could be manifested. Few articles (8 considered eligible for our systematic review) deal with this specific aspect.

The main protective factor that seems to be investigated by the limited research on this issue is social support. However, scientific publications do not seem to agree on this element, and the results seem non-homogeneous and univocal among the different cultures. Other possible protective factors could be help-seeking behavior and local/national services that deal with both assistance and information on the dynamics of abuse. This last aspect seems particularly significant for IPV prevention and the development of skills among the population during the entire life cycle. Regarding the risk factors that have emerged, the number of publications is greater. Similar to the literature on violence between couples, even studies on the elderly population show that older women are exposed to greater risk, while female-to-male violence is less explored. About this last result, we ask ourselves how much the “hidden number” affects the scarce findings about male victims of violence inflicted by their female partners. In fact, this factor implies an underestimation of the actual prevalence of the phenomenon (WHO, 2014, 2016) because of a men's certain reluctance to declare or denounce (and thus the tendency to minimize their involvement in violent dynamics between couples) IPV situations. For females as victims of violence between couples, an additional risk factor seems linked to economic conditions (low income and unemployment). The low level of education seems to be a risk factor although a high level of education is not a protective factor in itself.

#### Social-demographic variables

However, Guedes et al. (2015) question the results about the socio-demographic (economic and educational) status in their study of the role played by social support in the double role of risk and protective factors (Gerino et al., 2017). In fact, Guedes et al. (2015) point out that a low level of family support, loneliness, and isolation from the community increase the risk of being a victim of IPV; on the contrary, high levels of social support protect against the risk of suffering violence.

Other demographic variables that expose older people to a greater risk of IPV seem to be age (with “younger elders” aged 55–69 as the more exposed population) and membership in ethnic minorities, as well as cognitive and physical impairment. However, in the case of impairment, the need to study the causal relationship between the two factors (psycho-physical state and development of violent dynamics between couples) is particularly evident. Additionally, the analyzed studies show how substance addiction (particularly alcoholism) increases the risk of IPV. Depression also appears to be a risk factor, as well as a consequence of IPV. Even cultural beliefs, social values of reference (specifically machistic-patriarchal values), as well as racism and sexism, would have significant impacts on the manifestation of the IPV phenomenon.

#### Relational dynamics

Finally, the relational dynamics between couples, with reference to intrapartner dependence, the family, the partners' development history (specifically the intergenerational transmission of violence and trauma), and caregiving stress (in a manner often consistent with older couples, where one partner is affected by physical or mental illness), are identified as risk factors. For the development of the phenomenon of physical abuse, previous experience of verbal abuse would constitute a specific risk condition.

#### Methodological and application issues

All these findings, in connection with the outcomes and the enrichments that further research may bring, could help (1) to target specific public awareness and information policies, and (2) to offer helping professionals (such as psychologists, social workers, etc.) recommendations on how to best address situations of particular risk or vulnerability. Among the outcomes of the present review, the results showing how different disciplines are involved in the analysis of the phenomenon and the increasing number of published studies are noteworthy. Together with the use of the tools for analysis and detection that are already present in the literature, it would be advisable to prepare validated guidelines for screening and managing these complex situations.

Regarding the methodological issues of the analyzed studies, it has become evident that these lack a clear definition and specifications of the IPV construct as critical elements. In particular, the literature seems to be missing an important differentiation of the studies through an analysis of IPV in the light of the phenomenon's complexity and evolution. The construct (as defined in the research design), especially in quantitative studies on risk and protective factors, seems affected by the lack of distinction by type (as explained by the WHO), involving motivations at the origin of the phenomenon and the kinds of victims and perpetrators (see the models reported in the introduction section), in the direction of theory-oriented procedural optics (a clear explication of the theoretical framework).

On an even more general level, in line with the observations of McHugh et al. (2005) and Bell and Naugle (2008), studies concerning domestic violence between couples are affected by some critical issues of both theoretical and methodological relevance. In fact, current theories—both sociocultural (feminist and power theories) and individual (social learning theory, background/situational model, personality/typology theories)—fail to fully grasp the complexity of the factors involved in the phenomenon (McHugh et al., 2005; Bell and Naugle, 2008) and to be effective in terms of prevention and treatability (Bell and Naugle, 2008). For example, the feminist theory does not adequately explain women's violence toward their male partners, the presence of IPV between lesbian couples, and the lack of a significant relationship between sociocultural changes in attitudes toward the female gender (from more to less traditional) and IPV rates (McHugh et al., 2005; Bell and Naugle, 2008). This limitation implies the presence of bias in both the explanation of the violent interactions between couples and in the design of the variables to be included in the research on explanatory models, particularly in detecting antecedent or precipitating factors from a procedural perspective (Wilkinson and Hamerschlag, 2005; Bell and Naugle, 2008).

Greater attention is paid to contextual and cultural differences, but in our opinion, the need for cross-cultural comparative studies is increasingly evident, with emphasis on the issue of cultural minorities (e.g., in Western contexts). Nonetheless, always from a methodological perspective, it is noteworthy that no longitudinal studies deepen the knowledge on both risk and protection factors and how these vary over time. Again, it would be important to investigate which factors differentiate conflicting couples from those with IPV, as this would allow focusing on both the precipitating factors and the protection elements. In particular, in line with the findings of Roberto et al. (2014), it could be useful to deepen the differences between the situations where the abusive dynamic between couples is long-lasting (occurring in younger age and continuing up to old age), and those where IPV has its onset in the advanced phases of the life span. This would help both to increase the knowledge about the phenomenon and to design specific interventions. For this purpose and in general, it could be interesting to plan future research with mixed models (qualitative-quantitative studies) and with further attention to the peculiarities of the senior phase of the life span.

Taking as a reference Bell and Naugle's (2008) proposal, a useful explanatory model should include “multiple contextual units” (1101) that in turn involve relevant proximal variables. In fact, the authors' proposed model allows an even contextual analysis of the dynamics involved in violent episodes by using a micro- and a macro-analytic perspective and enables integrating the dynamic combination of multiple factors. This perspective, although still in progress, could be useful for a better understanding of the IPV construct involving the elderly, keeping in mind the necessity to elevate the complexity of the current interpretation (Bell and Naugle, 2008), as well as following what is indicated by McHugh et al. (2005) on the postmodern approach.

#### Limitations of the study

This review is limited by the availability of rigorous scientific publications on IPV in old age, particularly on both protective and risk factors involved in the development of this relational phenomenon in the life cycle. The study has several limitations. First, as mentioned in the methods section, we have exclusively considered the studies included in databases containing peer-reviewed international journals and published in English; this means that we have not considered possible studies in other languages or published in other types of journals (e.g., not peer reviewed). Thus, it is important to interpret the data concerning cross-cultural issues with caution.

Second, this paper is a systematic review (not a meta-analysis) related to protective and risk factors for IPV, without considering studies on intervention strategies and their outcomes. We have included only the scientific contributions that specify the constructs that are the objects of the studies, or we have analyzed in detail the elements relevant to the focus of this work, aware that this does not exhaust the comprehension of the phenomenon in its complexity.

Finally, this review is limited to the older population and does not consider studies involving participants belonging to other age groups. This choice of field is both a restriction on the application (generalizability) of the observations presented in this contribution and an analysis in line with the need to study more deeply into the peculiarities of the conditions of the elderly. A comparative study of similarities and differences in IPV manifestation across different age groups would be informative. This could be a desirable perspective and a compelling challenge.

#### Implications for future research and prevention projects

Considering the aspects highlighted so far, an increasing interdisciplinary approach in the study of IPV among the elderly is recommended. This implies the need for a greater complexity of explanatory models, adequately structured for the elderly, which take into account the complexity and particularity of this phase of the life cycle, including the elements of resilience and the fragility of this growing population. Such studies could provide important leads for policy and action to prevent IPV, starting from the precursors of violence that could be changed by preventive intervention, taking into account the research evidence that violent dynamics result from the interactions among contextual, individual, relational, and situational factors.

We consider it crucial to further explore risk and protective factors. Studies should differentiate between those associated with the “elder” onset of the phenomenon and those associated with IPV relapse/recidivism, monitoring the situation of the person entering the cycle of violence and the trajectory of the development of the abuse condition over time, including the factors of possible remission. Longitudinal and cross-national studies on the senior years and IPV should be conducted as well. Both these issues will offer the opportunity to create a protective network for people in difficulty and perhaps to create *ad hoc* services addressing this problem. To achieve these goals, we consider of primary importance the increasingly structured and constant cooperation among the professionals involved in planning scientific research and the practitioners of the community-based services. This interaction would allow mutual enrichment and critical attention to both the theoretical framework and the results of clinical work in specialized services.

## Author contributions

EG has been the responsibility of the design of the paper. EG and AC have been responsible for analyzing the systematic design and writing paper and in the creation of tables and images. LC searched for papers on databases and worked in their systematization. He collaborated in the process of paper writing. PB has been responsible for supervising the results and for the systematization of the papers. LR has the overall responsibility of the project and as supervisor of the paper, collaborated during the process of paper writing and in the finalization of it. All the author have been involved in the preparation of the discussions and conclusions.

### Conflict of interest statement

The authors declare that the research was conducted in the absence of any commercial or financial relationships that could be construed as a potential conflict of interest.
